# Molecular identification and functional analysis of *chitinase* genes reveal their importance in the metamorphosis of *Sarcophaga peregrina* (Diptera: Sarcophagidae)

**DOI:** 10.1093/jisesa/iead107

**Published:** 2023-11-28

**Authors:** Yakai Feng, Shiwen Wang, Fengqin Yang, Yanjie Shang, Fernand Jocelin Ngando, Jingjing Huang, Yadong Guo

**Affiliations:** Department of Forensic Medicine, School of Basic Medical Sciences, Xinjiang Medical University, Urumqi, Xinjiang 830017, China; Department of Forensic Medicine, School of Basic Medical Sciences, Xinjiang Medical University, Urumqi, Xinjiang 830017, China; Department of Forensic Science, School of Basic Medical Sciences, Central South University, Changsha, Hunan 410013, China; Department of Forensic Science, School of Basic Medical Sciences, Central South University, Changsha, Hunan 410013, China; Department of Forensic Science, School of Basic Medical Sciences, Central South University, Changsha, Hunan 410013, China; Department of Forensic Medicine, School of Basic Medical Sciences, Xinjiang Medical University, Urumqi, Xinjiang 830017, China; Xinjiang Key Laboratory of Molecular Biology for Endemic Diseases, Urumqi, Xinjiang 830017, China; Department of Forensic Medicine, School of Basic Medical Sciences, Xinjiang Medical University, Urumqi, Xinjiang 830017, China; Department of Forensic Science, School of Basic Medical Sciences, Central South University, Changsha, Hunan 410013, China

**Keywords:** Sarcophaga peregrina, chitinase, metamorphosis development, RNAi, vector control

## Abstract

*Chitinases* play a crucial role in insect metamorphosis by facilitating chitin degradation. *Sarcophaga peregrina* (Robineau-Desvoidy, 1830) (Diptera: Sarcophagidae) is a typical holometabolous insect and an important hygiene pest that causes myiasis in humans and other mammals and acts as a vector for various parasitic agents, including bacteria, viruses, and parasites. Enhancing the understanding of the metamorphosis in this species has significance for vector control. In this study, we identified a total of 12 *chitinase* genes in *S. peregrina* using bioinformatic analysis methods. Based on transcriptome data, *SpIDGF2* and *SpCht10* were selected for further functional investigation. The down-regulation of these genes by RNA interference led to developmental delays, disruptions in molting, and differences in cuticle composition during the pupal stage. These findings underscore the pivotal role of *chitinase* genes in the metamorphic process and offer valuable insights for effective control strategies.

## Introduction

Animals have evolved a protective biological armor to serve as the first line of defense against various environmental threats, including physical, chemical, dehydration, and pathogen challenges. The key component of this armor in mammals is the skin, while in arthropods, particularly insects, it takes the form of the cuticle (Zhao et al. 2021). The evolutionary success of insects, which represent the most diverse group of organisms on Earth, can be largely attributed to the development and adaptation of their cuticles (Zhu et al. 2016). And there are 2 essential substances in the insect cuticle: cuticular hydrocarbons (CHCs) and chitin. CHCs are essential components of the lipid wax layer and play a crucial role in limiting water loss and facilitating intraspecies communication (Chung and Carroll 2015, Blomquist and Ginzel 2021). Chitin, a major component of insect cuticles, also features in the internal structures of many insects, including the alimentary canal, tracheal system, genital ducts, and various dermal glands (Hegedus et al. 2009, Muthukrishnan et al. 2012). The cuticular chitin undergoes a tanning reaction to fortify it, thereby preserving the outer morphology of insects and safeguarding against mechanical damage, particularly during pupal development (Andersen 2010). Furthermore, the biosynthesis and degradation of chitin must be precisely regulated throughout the molting and metamorphosis cycles (Moussian 2010, Yao et al. 2010), with *chitinases* emerging as pivotal enzymes in these processes.

*Chitinases* belong to the family of 18 glycosylhydrolases (Arakane and Muthukrishnan 2010). The homologs of these enzymes have been identified and characterized in various species, such as 14 genes in *Bemisia tabaci* (Hemipteran) (Peng et al. 2021), 11 genes in *Agrotis ipsilon* (Lepidopteran) (Li et al. 2022), 19 genes in *Leptinotarsa decemlineata* (Coleopteran) (Chen et al. 2023), and 14 genes in *Aedes albopictus* (Dipteran) (An et al. 2023). Additionally, several *chitinases* were found to be essential for insect survival, molting, and development (Tachu et al. 2008, Zhang et al. 2021). Notably, the down-regulation of *Cht10* results in severe phenotypes, including wing differentiation failure, ecdysis disturbance, growth inhibition, pupariation failure, and death (Dong et al. 2020, Qu et al. 2021). Meanwhile, it was reported that *IDGFs* were essential for *Drosophila* fertility (Sustar et al. 2023). Despite extensive studies on *chitinase* function in numerous insects, its role in the flesh fly remains unexplored.

*Sarcophaga peregrina* (Robineau-Desvoidy, 1830) (Diptera: Sarcophagidae) ranks among the most prevalent flesh flies, with a wide distribution across tropical and subtropical regions in the Palearctic, Oriental, and Oceanian territories (Xue et al. 2011). These flesh flies exhibit a unique reproductive strategy, where adults give birth to offspring in the form of larvae on human or animal corpses, a phenomenon known as ovoviviparity (Majumder et al. 2014) and therefore considered of great forensic significance (Sukontason et al. 2010, Toukairin et al. 2017, Wang et al. 2017). Moreover, *S. peregrina* can cause myiasis in humans and other mammals, serving as a vector for various parasitic agents, including bacteria, viruses, and parasites. It has been implicated in the transmission of diseases such as cutaneous leishmaniasis (Miura et al. 2005, Lee et al. 2011). Understanding the regulatory mechanism of *S. peregrina* metamorphosis can provide valuable insights for pest control. Metamorphosis is pivotal to the evolutionary success of insects, yet its underlying regulatory mechanisms remain incompletely elucidated.

Our research commenced with the identification of 12 *chitinase* genes, accompanied by a comprehensive analysis of their structural attributes and conserved motifs. Subsequently, we selected 2 differentially expressed genes, *SpCht10* and *SpIDGF2*, based on transcriptome data, for an in-depth investigation of their functions during *S. peregrina* metamorphosis, employing RNA interference technology. Injection of gene-specific double-stranded RNA (dsRNA) resulted in a significant down-regulation of transcript levels. We employed scanning electron microscopy (SEM) and hematoxylin and eosin (H&E) staining to discern microscopic and internal structural changes, while gas chromatography–mass spectrometry (GC–MS) was employed to determine alterations in CHCs within the puparium. Our study unveils the pivotal roles of *SpCht10* and *SpIDGF2* in the metamorphic development of *S. peregrina* and underscores their potential as molecular targets for pest control.

## Materials and Methods

### Insect Rearing and Sample Collection

Adult specimens of *S. peregrina* were trapped with pork lung bait in Changsha, Hunan Province, China, and then employed pork lungs as a medium for larval rearing. Subsequently, we performed 6 generations of inbred crosses to reduce genetic variability. The adults of each generation were kept at 25 ± 1 °C and 70 ± 5% relative humidity with a photoperiod regime of 12:12 h light/darkness in an artificial climate chamber (GPL-250A, Tianjin Laboratory Instrument Equipment Co. Ltd., Tianjin, China). The new generation larvae were divided into 2 groups at 25 °C: one group was reared for *chitinase* gene expression pattern during the pupal stage and another was reared for dsRNA injection. When approximately 50% of the pupae were observed within 24 h, the *S. peregrina* pupae samples were randomly taken from all sample individuals. At 24-h intervals, 20 pupae were randomly taken from the rearing boxes until more than 5 pupae emerged, and these pupae were placed into a 5-ml cryovial and immediately frozen in liquid nitrogen and stored at −80 °C for the subsequent RNA isolation. Three biological replications of each treatment were collected for all samples. Pupal development lasted 9 days at 25 °C. A total of 540 pupae were collected.

### Candidate Gene Identification and Sequence Analysis

Amino acid sequences of *chitinase and chitinase-like* genes from *Drosophila melanogaster* were downloaded from the National Center for Biotechnology Information (NCBI) (https://www.ncbi.nlm.nih.gov/, accessed 28 December 2022) as queries to search against *S. peregrina* genome for screening *chitinase* genes in *S. peregrina*. The candidate *S. peregrinachitinase* genes were confirmed by searching the BLAST algorithm against the non-redundant protein sequence database of the NCBI. The predicted domain architecture of the *chitinase*-encoding proteins was predicted using the online website Batch CD-Search tool of NCBI. The theoretical isoelectric point and molecular weight were computed using the ExPASy online server (http://www.expasy.org/tools/protparam.html). The sequence alignment and phylogenetic tree were constructed using MEGA 11.0 with the neighbor-joining method and bootstrap analysis with 1,000 replications. Jalview software was used to conduct the alignment of amino acid sequences of the catalytic domains.

### Developmental Expression Analysis of *SpCht10* and *SpIDGF2* Genes

The pupal stage lasted 9 days at 25 °C, and the expression patterns of *SpCht10* and *SpIDGF2* genes during each day of *S. peregrina* were performed by quantitative reverse transcription–polymerase chain reaction (RT-qPCR). According to the manufacturer’s instructions, total RNA was extracted from the tissues of pupariation using the SteadyPure Universal RNA Extraction Kit. RNA purity and concentration were assessed by the NanoDrop 8000 (Thermo Fisher Scientific, Waltham, MA, USA). RNA was used as a template, and cDNA was synthesized by reverse transcription according to the instructions of the Evo M-MLV Reverse Transcription Premix kit (AG11728, Accurate Biology, Hunan, China). Specific primers were designed using the online website Primer3 (https://primer3.ut.ee/). All primer sequences are shown in Table 1. RT-qPCR experiments were using the SYBR Green Premix Pro Taq HS qPCR Kit (Code. AG11701). Each PCR was conducted in a 50-µl reaction mixture containing 20 µl of 2× SYBR Green Pro Taq HS Premix, 1.6 µl of each primer, 4 µl of cDNA template, and 12.8 µl of RNase-free water. β-Actin was used as the reference gene for the normalization of expression levels. Three biological replicates and 3 technical replicates were performed. Relative expression levels were calculated according to the 2^−△△CT^ method.

**Table 1.** Primers used in the RNAi and RT-qPCR analysis

RNAi	Gene name		Primer sequences (5'→3')
	*dsSpIDGF2*	Forward	TAATACGACTCACTATAGGGCCCGAAGAGGCAGACTACAC
		Reverse	TAATACGACTCACTATAGGGTGACAGACCACCTAAGCCCT
	*dsSpCht10*	Forward	TAATACGACTCACTATAGGGAAATACGGTTTCGAGGGCTT
		Reverse	TAATACGACTCACTATAGGGCCAGCTCTAGTGAAGGTGCC
	*dsGFP*	Forward	TAATACGACTCACTATAGGGCTACCTGTTCCATGGCCAAC
		Reverse	TAATACGACTCACTATAGGGTTTTCGTTGGGATCTTTGGA
RT-qPCR	*SpIDGF2*	Forward	CTCAATTTAGGCATCGCAACC
		Reverse	GCCTCTAAAATCACCAGCCTC
	*SpCht10*	Forward	CCATTGAGAGTGTACCCGAAG
		Reverse	TCCAAAGAGCGTGTACTAACC
	*Chitin synthase* (*CHs*)	Forward	CTTTCGTGGCTGCCTTCCGTATAG
		Reverse	GCGTAAGCCGTTGATAGGACCTG
	*SpCht2*	Forward	ATGTACTCGGTTCACAGGAATGCG
		Reverse	GCACCTTGACCAGGACGATACAC
	*β-N-acetylglucosaminidase*	Forward	CGTCCTCCTACTGCCCCAAAATTG
		Reverse	TCGTCACCATCTTGATGCCTTGTAC
	*Actin*	Forward	AAGAACAAGGTGAAGAGGGAC
		Reverse	TCGTCTTCATCGCGGAATATG

### Synthesis of dsRNA

To further study the gene functions of *SpCht10* and *SpIDGF2*, RNA interference was performed. The sequence-specific primers were designed in E-RNAi (http://www.dkfz.de/signaling/e-rnai3/idseq.php) based on the cDNA sequences of *SpCht10* and *SpIDGF2*, and *dsGFP* was used for negative control. Primers are listed in Table 1. The dsRNA of *SpCht10*, *SpIDGF2*, and *GFP* were synthesized in vitro by following the instructions of the T7 RiboMAXTM Express RNAi System (Promega, Madison, WI, USA). Amplicons were verified by DNA sequencing, and the integrity of the dsRNA product was confirmed by 1% agarose gel electrophoresis. The synthesized *dsSpCht10*, *dsSpChtIDGF2*, and *dsGFP* were dissolved in appropriate volumes of nuclease-free water, and the concentration was determined and adjusted to 2.0 µg/µl using a NanoDrop 8000 spectrophotometer (Thermo Fisher Scientific, Waltham, MA, USA). Samples were then stored at −80 °C.

### Injection of dsRNA

Using a Drummond digital microdispenser (Drummond Scientific Co., Broomall, USA), approximately 2 μl (4 μg) dsRNA was injected into the dorsal side of the second and third abdominal segments of post-feeding larvae. Then, all samples were exposed at a constant 25 °C, and the pupariation time, pupariation rates, the development time of the pupal stage, and eclosion rates were recorded at the same time. Morphological changes in the puparium were observed and recorded under a Zeiss 2000-C stereomicroscope (Carl Zeiss, Germany). At 24 and 48 h post-injection, 3 pupae samples were collected, and the RNAi efficiency of *SpCht10* and *SpIDGF2* genes was calculated by RT-qPCR, and the expression level variation of genes involved in the chitin-related pathway, including *Chitin synthase* (*CHs*), *Cht2*, and *β-N-acetylglucosaminidase*, was determined using the same methods. An independent sample *t*-test was used for statistical analysis. Asterisks indicate significant differences (*P < 0.05; **P < 0.01; ***P < 0.001). A total of 30 larvae were performed in each treatment group, and experiments were repeated independently 3 times.

### SEM Observations

To compare the morphology differences in pupal cuticles after *chitinase* knockdown, 5 specimens were obtained from each RNAi treatment group. Dissect the tissues inside the pupa with insect needles and tweezers and keep the puparia. The puparia were cleaned with water in an Ultrasonic Cleaner (Scientz, SB-5200D, Ningbo, China) for 1 h and rinsed with distilled water twice. Water on the puparia surface was adsorbed with filter paper and then allowed to dry at room temperature. Puparia were gently placed onto double-stick tape on stubs and coated with gold for 50 s in a sputter-coating apparatus. Subsequently, the morphological changes were directly observed under the SEM (Hitachi, SU5000, Japan).

### H&E Staining

To further determine the effects of *SpCht10* and *SpIDGF2* gene silencing on pupal metamorphosis development of *S. peregrina*, H&E staining was performed. Three pupae samples of each group were collected for the H&E staining at the later pupa stage after *dsGFP*, *dsSpCht10*, or *dsSpIDGF2* injection. The puparium was removed, and the morphological changes of the tissues of pupariation were observed and recorded under a Zeiss 2000-C stereomicroscope (Carl Zeiss, Germany) (Wang et al. 2018). The tissues of pupariation were immersed in a formaldehyde solution for 48 h and then transferred to 70% ethanol for 48 h for fixation. Then the usual procedure of dehydration, paraffin infiltration, embedding, sectioning, and H&E staining was followed (Davies and Harvey 2013). The internal histological changes of the tissues of pupariation after RNAi were observed and recorded using the automatic digital slice scanning and application system and Motic DSAssistant Lite (Motic, USA).

### CHC Analysis

To further determine the effects of *chitinase* on the puparium component, CHC profile analysis was performed. The puparia were cleaned in ultrapure water and blotted dry with filter paper. Then, each individual was immersed in 1-ml hexane at room temperature for an hour. Next, a syringe filter transferred the immersed liquid with a 0.45-µm aperture nylon membrane. After that, the liquid was dried under a vacuum and then dissolved in 100-µl hexane for GC–MS analysis. GC–MS (Agilent Technologies, 7890B-5977A GC/MSD), with a DB-5MS capillary column (30m × 0.25mm × 0.25µm), was used for the CHCs analysis. Equipment operation procedures refer to the previous research (Zhang et al. 2022). The *n*-alkane mix from heptane to tetracontane (C7-C40, 1 µg/ml, O2SI) resolved in 1-ml hexane was used as an external standard. MSD ChemStation Data Analysis F.01.03 was used to integrate the peak height, and only compounds with a consistent peak height percentage above 0.5% were included. Hydrocarbons were identified using a library search (NIST14) and the Kovats Index based on external standards and literature (Zhang et al. 2022).

## Results

### Identification and Protein Structure Analysis of *Chitinase* Genes

**Fig. 1.**
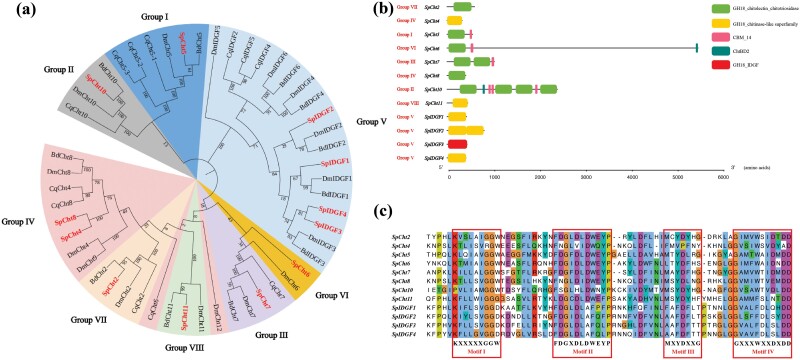
Bioinformatics analysis of *chitinase* sequences of *Sarcophaga peregrina*. a) Phylogenetic relationships of *chitinases* from different species. Dm, *Drosophila melanogaster*; Cq, *Culex quinquefasciatus*; Bd, *Bactrocera dorsalis*; Sp, *Sarcophaga peregrina*. The neighbor-joining method with 1,000 replicates of bootstrap was used to construct the tree. The values of the bootstrap are shown at each branch of the tree. b) Domain architecture of *S. peregrinachitinases*. The different rectangles represent different domains, from top to bottom as follows: GH18_chitolectin_chitotriosidase (NCBI accession number: cd02872), GH18_chitinase-like superfamily (NCBI accession number: cl10447), CBM_14 (NCBI accession number: pfam01607), ChtBD2 (NCBI accession number: smart00494), GH18_IDGF (NCBI accession number: cd02873), and lines denote linker regions. c) Amino acid sequence analysis of the catalytic domain of *S. peregrinachitinases*. Four conservative motifs are displayed using boxes.

In this study, 12 *chitinase* genes were identified in *S. peregrina*, consisting of 8 *chitinase* genes and 4 *IDGF* genes. All of these genes were renamed *SpCht* or *SpIDGF*, followed by a unique number. The length of predicted *chitinase* proteins ranged from 331 amino acids (*SpCht4*) to 5,483 amino acids (*SpCht6*). Correspondingly, their molecular weight ranged from 38.39 kDa (*SpCht4*) to 604.83 kDa (*SpCht6*), and the isoelectric point ranged from 5.43 (*SpCht6*) to 9.61 (*SpIDGF1*) (Table 2). Phylogenetic analysis showed that the 12 *SpChts* genes were classified into 8 groups (groups I–VIII) (Fig. 1a), specifically *SpCht5* in Group I, *SpCht10* in Group II, *SpCht7* in Group III, *SpCht4* and *SpCht8* in Group IV, *SpIDGF1*, *SpIDGF2*, *SpIDGF3*, and *SpIDGF4* in Group V, *SpCht6* in Group VI, *SpCht2* in Group VII, and *SpCht11* in Group VIII.

**Table 2.** Summary information of *chitinase* and *chitinase-like* genes in *Sarcophaga peregrina*

Number	Gene ID	Gene name	Protein length	Chromosomal localization	pI	MWs (kDa)
1	Contig64.87	*SpCht2*	602	Chr03	6.14	68.77
2	Contig17.201	*SpCht4*	331	Chr04	7.67	38.39
3	Contig8.186	*SpCht5*	560	Chr05	5.89	63.70
4	Contig180.1	*SpCht6*	5,483	Chr01	5.43	604.83
5	Contig83.98	*SpCht7*	1,036	Chr03	6.00	116.77
6	Contig17.202	*SpCht8*	412	Chr04	9.33	47.23
7	Contig11.188	*SpCht10*	2,411	Chr02	6.18	271.83
8	Contig80.29	*SpCht11*	453	Chr01	7.20	51.79
9	Contig1.823	*SpIDGF1*	429	Chr02	9.61	48.77
10	Contig1.824	*SpIDGF2*	812	Chr02	6.03	92.49
11	Contig1.825	*SpIDGF3*	439	Chr02	6.60	49.16
12	Contig1.826	*SpIDGF4*	414	Chr02	8.47	46.38

MWs, molecular weights; pI, isoelectric point.

Domain architecture analysis showed that all predicted *chitinase* genes contained at least 1 chitinase catalysis domain, such as GH18_chitinase-like superfamily (NCBI accession number: cl10447), GH18_chitolectin_chitotriosidase (NCBI accession number: cd02872), and GH18_IDGF (NCBI accession number: cd02873) (Fig. 1b). Additionally, *SpCht5*, *SpCht6*, *SpCht7*, and *SpCht10* contained 1 or more chitin-binding domains, namely ChtBD2 (NCBI accession number: smart00494) and CBM_14 (NCBI accession number: pfam01607). The results of multiple sequence alignments suggested that 12 *SpChts* have 4 conserved motifs: KXXXXXGGW (motif I), FDGXDLDWEYP (motif II), MXYDXXG (motif III), and GXXXWXXDXDD (motif IV), where X is a nonspecific amino acid (Fig. 1c).

### Expression Pattern Analysis of *SpCht10* and *SpIDGF2*

**Fig. 2.**
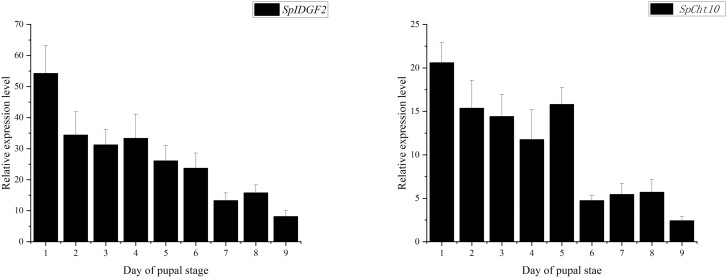
The developmental expression patterns of *SpIDGF2* and *SpCht10* during each day in the pupal stage (days 1–9) of *S. peregrina* by RT-qPCR. All data are shown as means ± SD.

We identified several genes that displayed significant differential expression patterns based on transcriptome datasets obtained from developmental stage comparisons during *S. peregrina* pupariation (Supplementary Fig. S1 and Supplementary Table S1) (Ren et al. 2022). Among them, *SpIDGF2* (gene ID: Contig1.824) and *SpCht10* (gene ID: Contig11.188) demonstrated a temporal decrease in expression trend during the metamorphosis, suggesting their potential roles (Supplementary Fig. S1 and Supplementary Table S2). To further confirm the differential expression of *SpCht10* and *SpIDGF2* during the pupal stage (days 1–9) of *S. peregrina* at 25 °C, we conducted an expression pattern analysis. The relative expression level of the *SpIDGF2* gene exhibited its peak on day 1 and its lowest point on day 9, demonstrating a gradual decline over time. Meanwhile, the expression level of the *SpCht10* gene remained relatively high from day 1 to day 5, with a noticeable decrease during the subsequent 4 days (Fig. 2).

### Effects of *SpCht10* and *SpIDGF2* RNAi on Metamorphosis Development of *S. peregrina*

**Fig. 3.**
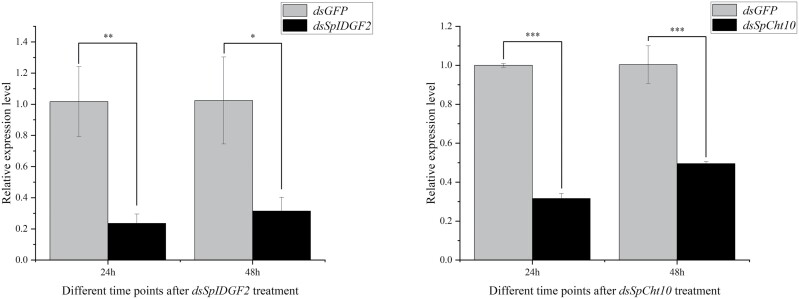
RNAi efficiency after injecting *dsSpIDGF2* and *dsSpCht10* for 24 and 48 h. *dsGFP* is used as the negative control for RNAi; actin is used as an internal reference for RT-qPCR. All data are reported as means ± SD (*P < 0.05; **P < 0.01; ***P < 0.001).

**Fig. 4.**
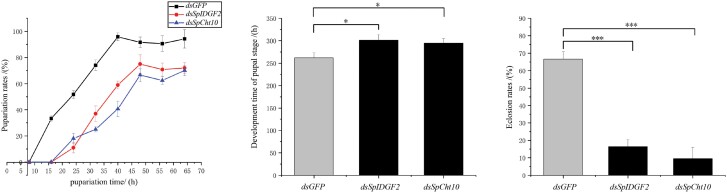
Effect of *dsSpIDGF2* and *dsSpCht10* double-stranded RNA (dsRNA) injection on the development of *Sarcophaga peregrina*, including a) the pupariation time, b) the development time of pupal stage, and c) eclosion rates. *dsGFP* is used as the negative control; all data are reported as means ± SD (*P < 0.05; **P < 0.01; ***P < 0.001).

**Fig. 5.**
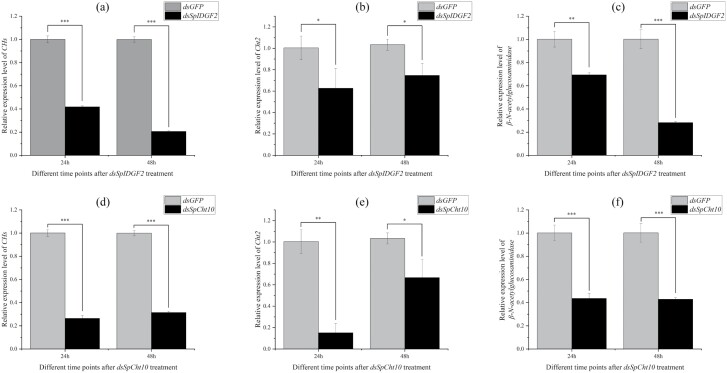
a–f) The relative expression level change of 3 genes *CHs*, *Cht2*, *β-N-acetylglucosaminidase* after injecting *dsSpIDGF2* and *dsSpCht10* on 24 and 48 h. a–c) *dsSpIDGF2*-injected group. d–f) *dsSpCht10*-injected group. *dsGFP* is used as the negative control for RNAi; actin is used as an internal reference for RT-qPCR. All data are reported as means ± SD (*P < 0.05; **P < 0.01; ***P < 0.001).

To assess the efficiency of *dsSpCht10* and *dsSpIDGF2* injection, RT-qPCR experiments were conducted. The transcript levels of *dsSpCht10* and *dsSpIDGF2* at 24 and 48 h post-injection exhibited a significant reduction compared with *dsGFP* injection (Fig. 3). Additionally, we recorded phenotypic indices to investigate their impact on metamorphosis development, including pupariation time, pupariation time rates, the development time of the pupal stage, and eclosion rates. Pupariation time was notably delayed in the *dsSpCht10* and *dsSpIDGF2* injection groups compared with the *dsGFP* control (Fig. 4a). Pupariation rates were 94.4% in control, 75.0% in *dsSpIDGF2*, and 69.4% in *dsSpCht10* (Fig. 4a). The development time of the pupal stage increased to 301.3 h in *dsSpIDGF2* and 294.7 h in *dsSpCht10*, compared with 262.0 h in the control (Fig. 4b). Eclosion rates for *dsSpIDGF2*, *dsSpCht10*, and *dsGFP* were 16.43%, 9.57%, and 66.63%, respectively (Fig. 4c). Furthermore, the expression levels of several chitin-related genes, including *CHs*, *Cht2*, and *β-N-acetylglucosaminidase*, significantly decreased after 24 and 48 h of *dsSpIDGF2* and *dsSpCht10* injections (Fig. 5).

**Fig. 6.**
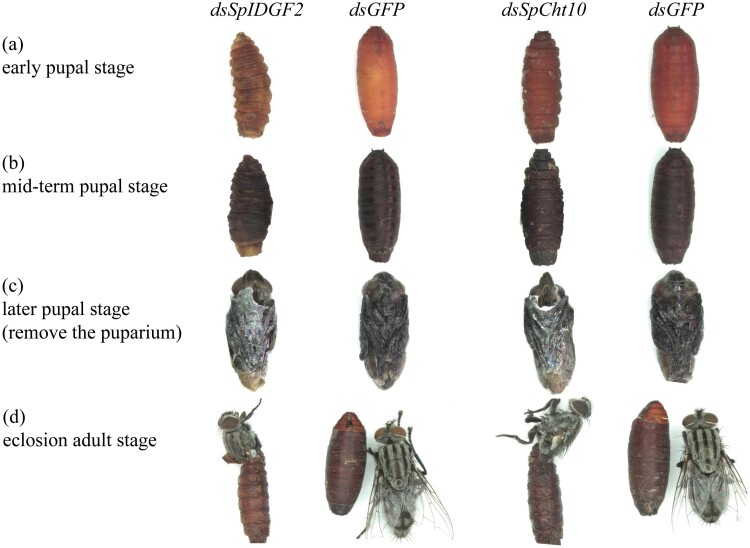
Phenotypic analysis of *Sarcophaga peregrina* after injected with *dsSpIDGF2* and *dsSpCht10*. The phenotypic changes of the experimental groups at different pupal stages were observed under the stereomicroscope, and the contemporaneous samples of the *GFP* injection group were used as controls. a) Early pupal stage, b) mid-term pupal stage, c) later pupal stage, the puparium was removed, and d) eclosion adult stage.

Observations of phenotypic changes in *S. peregrina* at different stages under a stereomicroscope revealed distinct differences between the *dsSpIDGF2* and *dsSpCht10* injection groups and the control group. During the early pupal stage, the control group exhibited a smooth and symmetrical puparium surface with shallow folds in the pupal segment, while the interference groups displayed rough, asymmetrical puparia with deep wrinkles (Fig. 6a). In the midterm pupal stage, some pupae in the interference groups exhibited reduced volume, a lighter puparium color, and increased deformity (Fig. 6b). In the later pupal stage, upon removal of the puparium, a transparent membrane was observed covering the bodies of *S. peregrina* in the *dsSpIDGF2* and *dsSpCht10* injection groups, which was absent in the control group (Fig. 6c). Although some *S. peregrina* can break their puparia, they were unable to fully emerge (Fig. 6d).

**Fig. 7.**
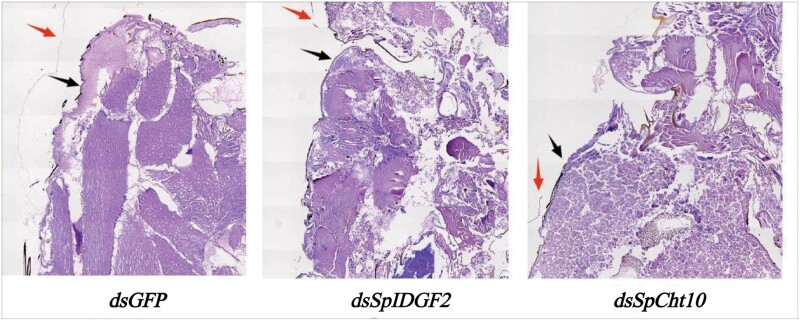
Histological observation of the tissues of pupariation at later pupa stage after *dsGFP*, *dsSpIDGF2*, or *dsSpCht10* injection by H&E staining. The black arrow points to the new epidermis, and the red arrow points to the old epidermis.

**Fig. 8.**
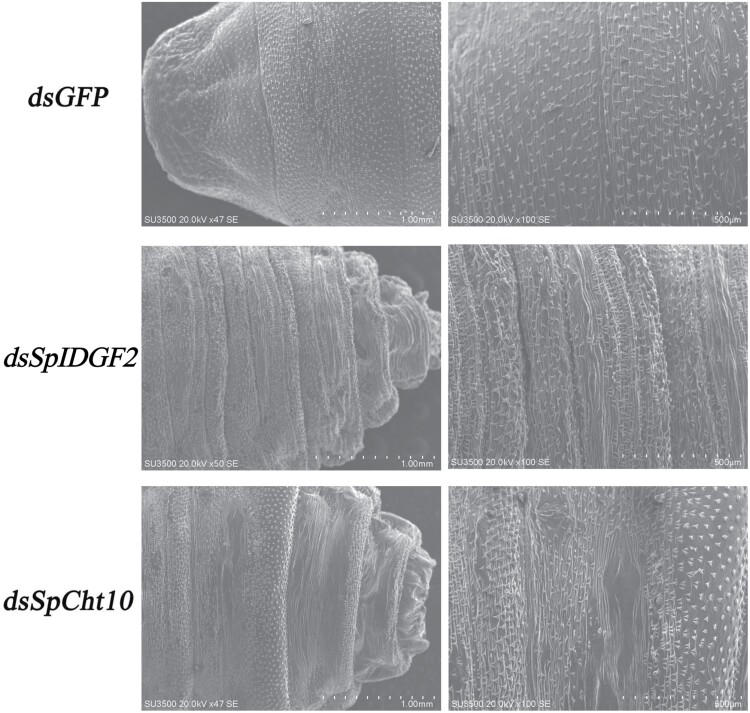
Morphology observation of the pupal epidermis at the later pupa stage after *dsGFP* or *dsSpIDGF2* and *dsSpCht10* injection by scanning electron microscopy.

Histological examination using H&E staining of tissues of pupariation in the 3 groups at the later pupal stage revealed that in the control group, the membrane had detached from the outer epidermis of the *S. peregrina* body, while it remained tightly connected in the interference groups (Fig. 7). SEM results displayed triangular, evenly arranged intersegmental spines on the puparium surface in the control group, contrasting with crowded, mixed, and disordered spines of varying sizes in the experimental groups (Fig. 8).

**Fig. 9.**
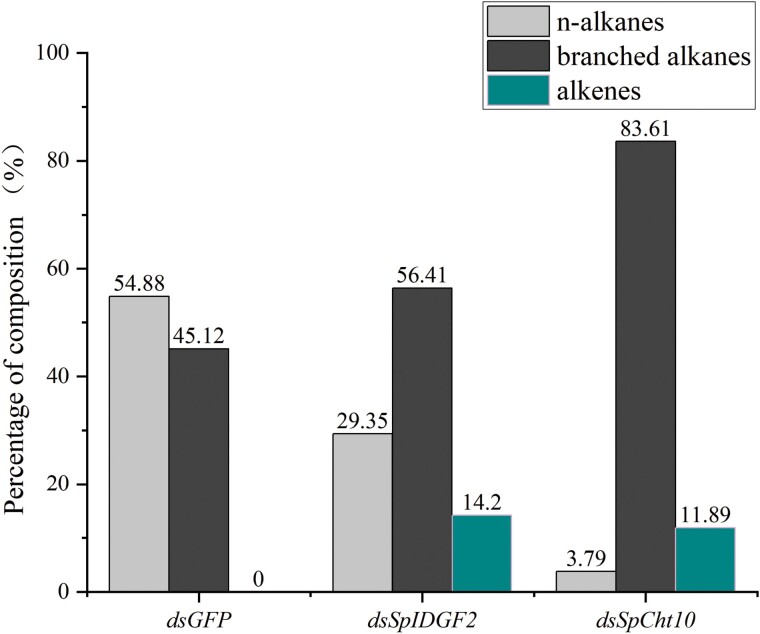
Percentage of puparium hydrocarbon composition of *Sarcophaga peregrina* after injected with *dsGFP*, *dsSpIDGF2*, and *dsSpCht10*.

Figure 9 illustrates the differences in puparium composition between the RNAi-treated groups and the control groups, with the CHCs detected given in Supplementary Table S4. GC–MS analysis identified a total of 48 CHCs in the puparium of *S. peregrina* after *dsSpIDGF2* injection, including 15 *n*-alkanes, 29 branched alkanes, and 4 alkene compounds with carbon chain lengths ranging from C14 to C35. Meanwhile, the *dsSpCht10*-injected group had 27 CHCs, comprising 4 *n*-alkanes, 20 branched alkanes, and 3 alkene compounds with carbon chain lengths between C17 and C33. The control group contained 36 CHCs, consisting of 16 *n*-alkanes, 20 branched alkanes, and carbon chain lengths ranging from C12 to C35. The *dsGFP* group exhibited *n*-alkanes at 54.88%, while the *dsSpCht10* and *dsSpIDGF2* groups had 3.79% and 29.35%, respectively. For branched alkanes, the *dsGFP* group had 45.12%, whereas the *dsSpCht10* and *dsSpIDGF2* groups had 83.61% and 56.41%, respectively. After RNA interference, alkenes were present at 11.89% and 14.2% in the *dsSpCht10* and *dsSpIDGF2* injection groups, respectively, but not in the control group.

## Discussion

Holometabolous insects undergo significant morphological and structural changes during the pupal stage, which appear to be a response to environmental adaptive evolution. During this period, the insects require a hard exocuticle to ensure the security of their internal changes. Chitin, a major component of the insect epidermis, plays an important role in pupal growth and development. *Chitinases*, a diverse group of enzymes, catalyze chitin degradation through hydrolysis (Kramer and Muthukrishnan 1997). They show significant variations in enzymatic properties, domain organization, and size (Merzendorfer and Zimoch 2003, Zhu et al. 2008, Arakane et al. 2009).

In this research, we identified 12 *chitinase* genes from the *S. peregrina* genome, which were classified into 8 groups based on the amino acid sequence similarity and phylogenetic analysis (Fig. 1a). Notably, *SpCht4* and *SpCht8* were located closely in the genome, suggesting a gene duplication event, a phenomenon also observed with *SpIDGFs* (Table 2). Three chitinase catalysis domains and 2 chitin-binding domains were found in *S. peregrina* (Fig. 1b). Analysis of domain architectures showed that *SpCht7*, *SpIDGF2*, and *SpCht10* contained more than 1 chitinase catalysis domain, suggesting potentially heightened catalytic activity. Furthermore, the presence of chitin-binding domains in *SpCht5*, *SpCht6*, *SpCht7*, and *SpCht10* suggests a tighter anchoring mechanism for enzyme–substrate interactions, facilitating the hydrolytic process catalyzed by the catalytic domain (Boot et al. 2001, Arakane et al. 2003). The degradation of chitin is a dynamic process that requires a coordinated action between both domains (Arakane et al. 2003). Therefore, *SpCht7*, *SpCht10*, and *SpIDGF2* would be priority candidates for use as targets in controlling *S. peregrina*. Additionally, the “DWEYP” sequence within motif II is considered a key signature, and the residue “E” is essential for catalytic activity (Fig. 1c). Therefore, for genes with only one domain, such as *SpCht4*, *SpIDGF1*, *SpIDGF3*, and *SpIDGF4*, their catalytic ability might be inactive.

*Sarcophaga peregrina* undergoes molting by the time they enter the pupal stage and prepare for the next molt in the later pupal stage, and the expression of these 2 genes in the pupal stage is consistent with this pattern (Fig. 2). Employing RNAi technology, we effectively down-regulated the transcription levels of *SpIDGF2* and *SpCht10* in *S. peregrina*, as confirmed by subsequent RT-qPCR experiments (Fig. 3). This down-regulation significantly impacted pupal stage development, leading to prolonged pupariation times, reduced pupariation rates, extended pupal stage durations (Fig. 4), and pupal malformation (Fig. 6).

It has been shown that the biosynthesis and degradation of chitin maintain a dynamic balance, and the content of chitin changes periodically with molting (Moussian 2010, Yao et al. 2010). To further confirm the impact of *chitinase* knockdown on chitin synthesis and degradation, genes involved in these pathways were selected for expression pattern analysis (Fig. 5). Chitin synthase, a critical enzyme in the final step of chitin synthesis, plays a vital role in insect survival, ecdysis, fertility, and egg hatching (Zhang et al. 2010, Ullah et al. 2019, Wang et al. 2019, Yang et al. 2019, 2021, Ali et al. 2020, Yu et al. 2020, Zeng et al. 2022). Additionally, chitin degradation relies on the cooperative action of *β-N-acetylglucosamine glycosidase* and *chitinase*. Therefore, the expression levels of *CHs*, *SpCht2*, and *β-N-acetylglucosaminidase* were evaluated following RNAi treatment, affirming a tangible impact on chitin synthesis and degradation (Fig. 5).

Furthermore, the RNAi treatment limited the ecdysis of *S. peregrina* during the pupal stage. Upon removal of the puparium in the later pupal stage, it was observed that the old epidermis within the tissue of pupariation failed to completely molt, remaining attached to the body surface (Fig. 6c). This incomplete ecdysis hindered the emergence of the new epidermis, a phenomenon corroborated by the results of H&E staining (Fig. 7). Notably, previous studies have reported a connection between *chitinase* and ecdysis. Tachu et al. (2008), Zhang et al. (2021), and Li et al. (2022) documented instances where down-regulation of *chitinase* inhibited larval molting. Similarly, in *Ae. albopictus*, suppression of *AaCht10* expression resulted in abnormal pupal molting and increased mortality (An et al. 2023). The increased mortality was also observed during this experiment, although we did not record it explicitly in this research. In summary, it is believed that *IDGF2* and *Cht10* genes play a crucial role in the molting process of *S. peregrina* during the pupal stage.

Finally, the down-regulation of *SpIDGF2* and *SpCht10* also exerted a notable influence on puparium formation, as evidenced by the results of SEM (Fig. 8). The changes in intersegmental spines on the puparium surface due to dehydration and shrinkage were similar to those observed in *Liosarcophaga dux* (Sukontason et al. 2006) and *Liopygia cultellata* (Ubero-Pascal et al. 2015).

It is noteworthy that changes in phenotype are often accompanied by modifications in internal composition. Therefore, in this study, CHCs were analyzed by GC–MS to reflect the changes in the internal composition of the puparium. The results of the CHC analysis show significant differences between the *dsGFP* injection group and *dsSpCht10* and *dsSpIDGF2* groups. Chitin and CHCs are 2 different biologically active substances in insect cuticles, but both play important roles in insect growth, development, and physiological functions. However, there has been no research on the connection between the two. Our study represents a novel attempt in this regard, potentially offering valuable insights.

## Conclusions

In this study, we identified a total of 12 *chitinase* genes in *S. peregrina*, each displaying distinct physicochemical properties. Knockdown of the *SpIDGF2* and *SpCht10* genes exerted significant effects on pupal development, ecdysis, and the structural composition of pupal cuticle compounds. These findings underscore the pivotal role of these genes in the metamorphic process and offer valuable insights into the control strategies.

## Supplementary Material

iead107_suppl_Supplementary_Figures_S1

iead107_suppl_Supplementary_Tables_S1

iead107_suppl_Supplementary_Tables_S2

iead107_suppl_Supplementary_Tables_S3

iead107_suppl_Supplementary_Tables_S4

iead107_suppl_Supplementary_Tables_S5

iead107_suppl_Supplementary_Tables_S6

iead107_suppl_Supplementary_Data

## Data Availability

The data presented in this study are available on request from the corresponding author.
