# Supplementary figures and images for: Molecular identification and functional analysis of chitinase genes reveal their importance in the metamorphosis of Sarcophaga peregrina (Diptera: Sarcophagidae)
Source: J Insect Sci. 2023 Nov 28;23(6):10. doi: 10.1093/jisesa/iead107 (PMC10684050; doi:10.1093/jisesa/iead107)

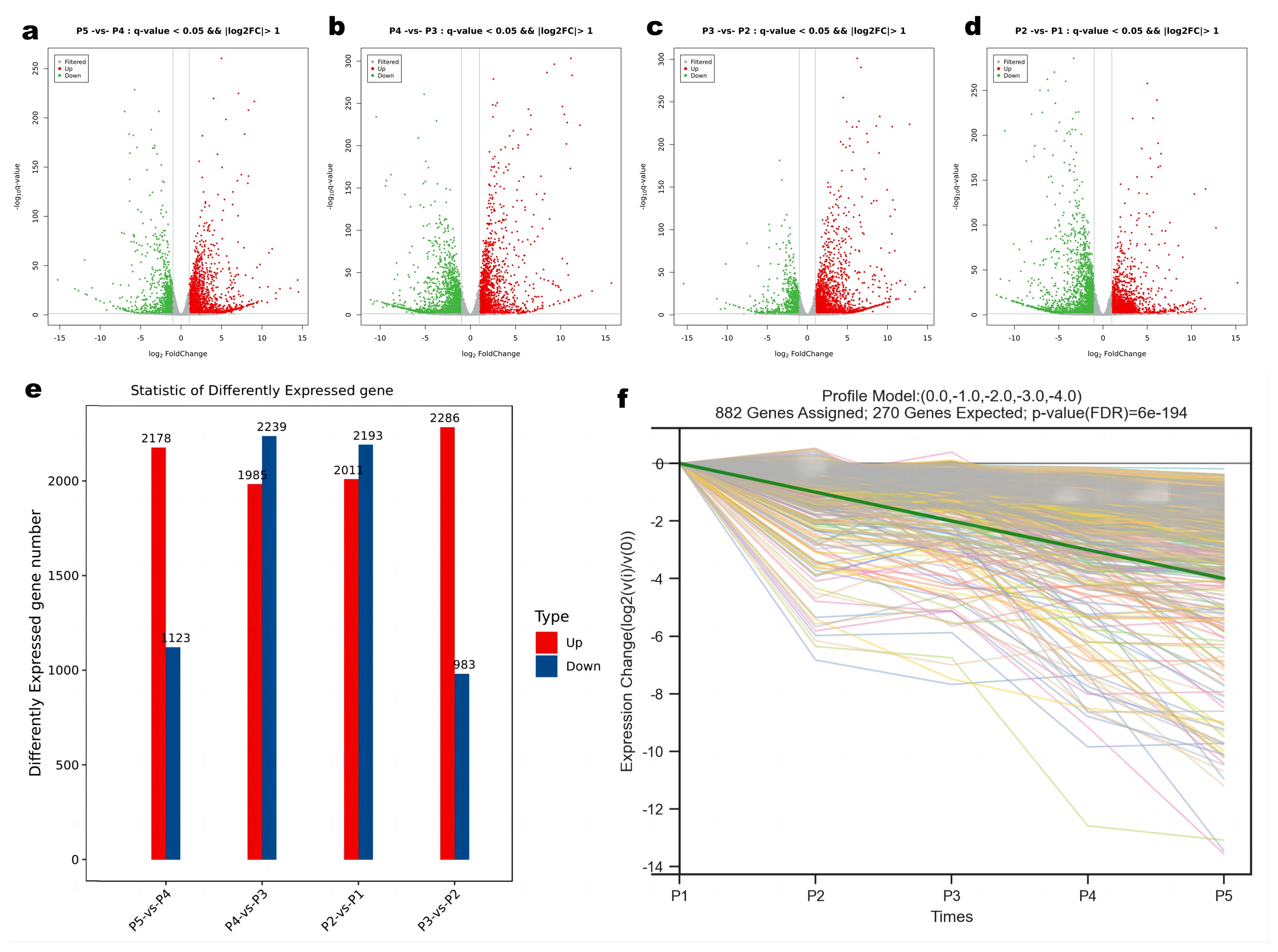

Supplement: iead107_suppl_Supplementary_Figures_S1 [file iead107_suppl_supplementary_figures_s1.jpeg]
