# Supplementary material for: Molecular identification and functional analysis of chitinase genes reveal their importance in the metamorphosis of Sarcophaga peregrina (Diptera: Sarcophagidae)
Source: J Insect Sci. 2023 Nov 28;23(6):10. doi: 10.1093/jisesa/iead107 (PMC10684050; doi:10.1093/jisesa/iead107)
Supplement: iead107_suppl_Supplementary_Data [file iead107_suppl_supplementary_data.docx]

**Supplementary Data- 1**

The sequences of chitinase and chitinase-like proteins in *Sarcophaga peregrina*.

>SpCht2

MTLVSPSLTSWPLCLEIKLNASSLQFNKLSRRSNSFAYKKVTRTVFYDINVEMQSSYQYIGGSILLLWNRIEKLKSFLKTLEYWKIKLNLHDENLCLLINKSEVEHQHNGITALIPTTPSILRIYICTRFTGMRFRPMHGKTVVCYISTWAVYRPGQGAYSIDNFDPSLCTHVVYAFAGLDPQQSAIKSLDPWQDLKEEYGKGGYERLTGLKRTYPHLKVSLAIGGWNEGSKNYSNMVADPMLRGKFVKQVTSFIRKYNFDGLDLDWEYPTQRGGAPHDRENFVLLCKELREQFNPHNLLLTSAIGAAKNVIDQAYDVRQISRYLDFLHIMCYDYHGSWDRKVGYNAPLTAQNGDVLSVQFTIDYLIKLGAAPEKIVMGVPFYGRTFRTPLDGNIDDDTDGTAFQGPFTREDGFLGYNEICRILSNKTSGWTTMWDPETSQALARSEKDVFTGLVEVVTFDNSRSIANKVKFAVDRKLAGIMVWSIDTDDFLGDCEPETDIYEDFLHFKHLDLALPHRLSNNYPLLRTINEAMPLAIEETKHKHDHPHRHDWDDNQEPDVSDNEIPHGSVEDHKGDSNKACSSKQAYTYFITIIVGILSLVL

>SpCht4

MDPSLCTHLIYEFCKLQQDGNLIEKNSQLDLDVNKGLGNYKKFNNLKQKNPSLKTLISVRGWEEESHNFSIVAADPKKRATFLQSSTEFLQKHNFNGLVIDWQYPNEYHELKNNDWENLAIWLKELSDGFKLYGYLLVIRTPKPTSMNNISELEKQLDFLIFMVPFNYGSIKFVTISSPLYAGPADQDEQARQRNFDAIAQFWLRQGTPRHKIIMSIRFYGRSFTLENSQNHSVGAPRNGSGIKGPYTREPGFVNYNELCEIFKSNDSAWHLEWESKQMVPYAYHDNQWIGYENERSVALKVDYVKKHNLGGVSIWSVDYADFRGVCGAKR

>SpCht5

MGIALTVSPTKASEQSARIVCYFSNWAVYRPGLGRYGIEDIPVDLCTHLIYSFIGVDDKSWEVLIIDPELDIEQNGFRNFTQLRQTHPQLKLQIAVGGWAEGGSKYSQMVASREKRQSFIRSIVNFMKKYDFDGFDLDWEYPGATDRGGSYGDKDKFLYYVQELRRAFEREGKGWEITMAVPVAKFRLQEGYHVPELCELLDAVHAMTYDLRGNWAGFADTHSPLYKRKHDQYAYEKLNVNDGLALWEDMGCPANKLVVGIPLYGRTYTLSSSNKNYNMGTYINKEAGGGAPGPFTNASGFLAYYEICTEVKDKANGWTVEWDEEGMVPYTYRDTQWVGYENEQSVQIKMDFIKIKGYAGAMTWAIDMDDFHGLCGRENALMHIIHDNMKDYMVPEPTRETTPRPEWAKPPATPPNPDEGSLVMLETTTRKPKPMTTKRPTTTITTTKKSMKTTKKPPTSSKPVVTTTMAAPAKEPATESTEVISPIEPMEPLDVDCAHRDFVPHQDCRKYYRCVHGKPVEFQCKEGLAFHTVSNVCDWIENSDRYYCTRLKDKQMGNNA

>SpCht6

MDTICPDKHLMYLSSIFLQMNTKIDASGVRSDGRVVCYYTNWSVYRPGTAKFNPQNINPYLCTHLIYAFGGFTKDNQMKPFDKYQDIEQGGYAKFTGLKTYNKQLKTMIAIGGWNEASSRFSPLVANAERRQKFIKNILKFLRQNHFDGIDLDWEYPAHRQGGKPGDRDNYAQFVQELRAEFERESEKTGRPRLLLTMAVPAGIEYIEKGYDIPKLNKYLDWFNLLTYDFHSSHEPSVNHHAPLYSLEEESEYNYDAELNIDYSIKFYLNAGADRDKLVLGIPTYGRSYTLINEESTEIGAPAEAPGEQGDATREKGYLAYYEICQNIKEDPEWTVVQPNPNAMGPYAYRRNQWVGYDDEAIVRKKAQYVVENGLGGIMFWAIDNDDFRGICTGKQYPLIEAAKEAMLDALGFGINEVSKPNAPQKPSRSRSRENSASTRNRITTTEHKLTYAERKGSTKRVQTVQQSSSRSKQQTSTTEAPIKLTEVEGSSLYIGGRTTTPPPPTTPDPGTDFKCEEEGFFQHPRDCKKYYWCLDSGPSGLGIVAHQFTCPSGLYFNPAADSCDFARNVPCKTKKSTTAAPITTTAGTTTTTTPRPNRLTAATSRPTTFFRTTPRTTTTTTTEASIEEDIEYEEEENSAPSPFAKKSKGSDKDAEEDPKVIKELIELIRKVGGLDELEKHLLRKEDGTISIKENSSSSSSSSLTATTPSTISKSLYEKVLSKPNALNSFRNRFTSTSSFRTTKTQTKTQDNAGAEVEIEETHETDNKSSGATTGSNKYSSVLRGNSRQGPQNEGISKLSEFDGFLKEKKQYITINRNRGGTKKTSGTEEGEEVQVEEEEEEISKTKSDSLTQEEQEEYDVKTTRKAFSSATPSYSTIRRTRPSTTTTEANNDEFEDAEEKEVTERKSYSTLNRSRSRATTTTETPDVVEVTAGSTRYKYLERTRPTRPTTESSDKIAEEEDAHNTEVEDDDEAKTKENTVHFKAVNIENTRETQQQQQQQEITTNTQRTYASIGRRTTTSTPDTETTTAGAAKTSTTTTTTAAPTTTSTTETPITTPLTIIETTTPDSILLTTNIPITTTTTTNPTTISTTSTTITTTTSRTTSLPNGEDVLGTSNTTPSVPNEINSDISETLDSSLHLNSLLIESSSTSSAAPTPIPTTESGLVTNEQEIDNSHTTTTEATISATTATTVLNGNDKLTKTADTDEHEEVNLVSKQKNKTLYNTRTRTTTTTTKTLPTITTTTNTNSDIETTTTTTSPTNAKKTLGVRRQQQVLLNRSSQTKTITTDTPNPTTTTTESVNNDFLGTVSSPRPFGFPKRRTRPSINAAGTTTATTTTDEEVVKSTEESLITKQKASTKLNAAANSQTKVSSHNTILSETTTTTTNTTNLHDAQTSSQQELLTQQDESEVTSTEHHNSKDTKVDALNQHVVVAINGSEGINDSLRHVNNVVLNLNNSNSTSNSNKTVQGSEHDNESSVLNNKFKYSSVTRGQSSKTGTEQENEHESESSGLKYSSVNRGQTVYKGTEENENENSGLNIKSKYSSINRGETVYKGTEQTESENSGLNNKFEHSSVIRGQSSKTATEQGNEHDNESSVLNVKSKYSSITRGQTSKSETQQGNEHDNESSVLNNKFKYSSVTRVQTSKTGTEQVNEHESESSGLKYSSVNRGQTVHKGTEENEKENSGLNIKSKYSSITRGQASKSETDQGNEHNNESSALNVKSKYSSINRDETVYKGTGENESENSSLNNKFEYSSVTRGQSSKTSTEKVNEHESESSALNVKSKYSSINRGETVYKGTGENESENSSLNKFEHSSVTRGQSSKTVTEQGNEHEGESSGLNIKSKYSSVNRGETVYKGTEQTESENSSLSNKFEHSSVTRGQSSKTATEQGNEHDNESSVLNIKSKYSSITRGQTSKSQTEQGNEHDNESSVLNVKSKYSSITRGQTSKSETEQGNEHDSESSSLNNKFKYSTVTRGQTSKTGTEQENEHESESSGSINRGTLTSTENIPYKAIVRDNGGIQLTEDQLSGFIKNAGEEISRKTYQAISRGGQVIQSENTEINEKDHYHINALNTLDVTEIESNRNANKEDATLEKSVAIEDNAATEKLSTLEASTTTTTSTTTTTTTEEPTTEEPTTASVLENTSSTEAAIETTTVPTTTATESTTRRSFSKTRVTTATSNADSETEVEKSVRKGTFNANTQGKKEEETTTLTGRRRTVYKRPTVTTQEGNSQNTNEKTETQTNFRRRTILRKKPSVIVQTETEIDETKQKFDRGSLVSLESESSQDGIETSTRANLPRATYRPRDKGDLSSLLALDLAKKSGNKATLTFGNGKRRRPGQSGLQVSETEDDSETTVRIGGSAQRRPGQSGLQVSETEDDSETTVRIAGSAQTFGNGKRRRPGLSGLQVSETEDDSEANVRIAGSASDDLVSNEESITTATRKKIFGSQGVKSKTEIKGETASEVAGNKIRTKITFGNTGKVTTETGNANNLAANGQQSTTQTVNGRRRVIVRKRIQAGQALRTQNANSVESSLVSNSNINNSESIVNQNGKAQIGLSNSRFSSFASKSKSNSESESVLGTETEDSEAVSEGEIKNDAASEVDVKDAKVTGSEKKFSYNSLNRKNAYANSRRVVGSQTVEEDSENTESVVRTANRATLNKVRNSSNSVRSQSQDSSRKSAYGNRSGSLNKKKHETTEEVEILEDGSKRIKTTFYETVNTGGQVKIIKRTKIKTIIKQEPEYIDETEDITPTTTTRRPLRSRGSAKFQPDDLSSLLALDFASKSNRHEQNDRSLTKHRRKVIRKPAIVKEEEFVEEIIEDLPTRPTRVPTTTEASQIISNRIKPTRPATTEANLTSIVESDSGLKVAIGEEKSVLSTNKRKFGIKRPTIVATKSTIQSTVVNETGKDFTTTRRKFAFKRPLGTGQTITNTEQITKTVITQTEQTNGNGNVVGGNKFINRFKSKSITTNKDEETTTGSLPTTSIVTESVTLETTNPVPSTEHTTETLEKSDDITNGLANVVNTLKTATEVETETKTNILKGKGFTKDITLDLIEAVSGLKTQAEKVTETEVSDAIETKNEIIAGKAFGQDLSLPIYHRRKYYQYYKDSPITYISPATGRSETEQTNVNVDQQIHDVFNVSNEHLPENHENNDAVIAVKAVEQAQQINSELSHFLLKTPGSNETSETSATEITQEPAASTEATKNFELDLPAEKENETTFGKLNRPLIKSTTEKALVDQETNTKSFNRLRTTTLKSLKETTNENKETVTTLRLNPLLANRSRVSLNRKTTDASLTTDTPVTERSTNRLIRYRATTPQSNEVENEDEQTSTLRSIGSKTFPTRRSFGGVNRLRITTTETSESTTEDDDEDTTNIEVSSKRTLVTRKPFGGFKRPTTTTTEALAEEPESTIENDDEEITNEKVSSKRTLVTRKPFSGLKRPTTTTTEALAEEPESTIENDDEETTNKEISSKRTFVTRKPFGGFKRPTTTTTEALAEEPESTIENDDEETTNKEVSSKRTLVTRKPFGGFKRPTATTTEALAEESESTIENDDEETTKNVAPALRSFATRKPFGTFNRPTATTTEASAEITEDTTYNDEEETTETVATPKRTLPTRRPIGGLNLSTSSTTEASTEKSEVIVGNKDEPTIEDDTYTTTTTDGLTDENKSTSGNDDENTSEEVASTTPFTDVNLSTSKTLTVDLESTIEQTVTKVTTTETLGSSNQPTTATAEPSTEQSESVIENEDEEYENDEDEEKIVDFIDESNEDEQVDEENYDATILTNKNENKLANPELALKVPSSLHLTTRNRVHLGPNHDNENLKNLLNAKTKFHIAANQHADTSRTSELTTEVNNKEVAFNENTNTNANILNHSSFDTNRSSSTVANYLNRIKINNLSSKFTNTAPSHTVDDQQKMKINFKLVSSDVNENMNDNDNEFDKEMNEEEEEEVNEFEVSNGRDKSYDNDNNNDDDNDNDDDVNKGDDNDSKRRFQKYQFQRRTTTTKATTTTTRTTTTTTEKPTAYKKEEIQDAIAKTQVNVNNEGNESEISTNIPEKTTAPTTTTTTESPSSPAAPLTRRLKVLKLKRPVVVSSNNNTNITANTTITANTATATAPASDGSATNLNASHTDTQNISSTNHESQTDDPLLKLRNKVKQDLAKGSQEEKGVLSTRFKSSQSSGTEQQPHVKDDPFLKLRNKVKQDLAKGNQVEGVLTERFNSLKSNQTEVDDPLLKLRNKVKQELAKGEQEEGVVNLKLKKLLETKNTTSGINYRESVSTTTTTAPRTKPTFRRKLVAKRPYIPTKPPISALRTTTIKIPTTKGKKKYVRRKFGRFHPFNASNRNTGEGFIRKDPRGNILPGTDRFKIENSAGGHSGDGQDYDEYDEEDENEGYNEEEEEEEEQEVEKEEIKPVQAIKPVINRPPGLFNRPSITILKKPTEQSSKEQTSAEEEEEDEEDETDDEEEEDKEEAEDAEEEEKENDSETIGKTEQTPIARPTYKPKDNRIPPGARPALTTTTINKKPNTPSRVANTPYRPDSGLQSATTQGNRGNRFGSSSSSSRPTVKPRVVNRPNGVSVPNLTIRPVANDFEPTTVLIPTKPAPFINPNTRAYERKYTGPTTESTGDLIGDNNPLIEDLNIEALNARNKKIFDINSKKHTTLKPKVVTPLIQLTEDTAQQILNTENENDEGYQSSVNGNGSSTITTTTTITTNTITQQQQEQEHEHEHEHQQQEHEQSTTQDNTNNNTGTQDTQDENLSNPNNLNEISSQEAVYTTTPTPSTTLLHIFTIADAEEHVTRNPLEDGYQVVPRLIVERNKPKHKVVEINRIVEVNSKEEKLRRKSKANIAIPVEGSETFKVESLPHIEQLGEISVVKYVHQVDGSDITIEGRSTVTDYTPTEPTVPDRPQPPIRYTLPDGSVGVEERSGKALLPEVLREAIETSTISLEGLFENARKGKLLNTVEEVTVSPGQIETTTEKSATGLRPTTTANSNGFTVTRRPVLSVRRRLINLSTAATTISATSSPTVTTTPNLTTPPVSSSTLSDDYTTTSKFKRLRTNRPPTINSAAVDITTTVTNAATASFKLNSQQKKKSSETSSNDLRLASSSLTSSSADNDITARQSQSSAALRRKYQARRLTTPTSSNTNSDGSATEPPRTQNPLFKRRFSLTTFSPLSSKTVLPSSSLSKATTTTSASASTSPLATATLRTGIDSLTTTVYVDGLENDEATDQVAKSSIHTSRFNQINGRLHIDDDDDEELETLDGGGNNGGGVEDLSHVVNTVKTITTLSPATKHTELPSPLQQQQQQIYSKYNFEDVITRRMDGTGTVAKLPASTTTATSTTPTTVIKKRKMIIKRRPQKATPSSEVAASVSSSKRRKPNKYQEPFSRLNSNQEKVVAKPSQQSARTYRPNLDYDYYDDEDVRLVGNKNDQQLKVILHGGGIIECLDQGNFPHPLSCRKFISCAKFETGGVVGWEYTCPKGLSYDPVGGMCNWAAGLGCKE

>SpCht7

MVVGNFKPYTWEKSGHRIWLSLPPRIFKFVFLICLILVLITQTTDSAQTKTRRRLRRPTSAPEEEVSSSSVASVRSTRLSGKKSTENSADKRIDQESSPSTTVTRSRLRSKSKLRNSASSDVSSGLIASGSSLKGKKTKADDGGKKIVCYYTNWSQYRPKIGKFVPEDIPADLCTHIIFAFGWLKKGKLSSYESNDETKDTVPGLYDRMMTLKKANPKLKILLALGGWSFGTQKFKDMSATRYTRQTFIYSAIPFLRKRGFDGFDLDWEYPKGSDDKKNFVLLLKELREAFEAEAQELKRQRLLLSAAVPVGPDNVRGGYDVPAVASYLDFINLMAYDFHGKWERETGHNAPLYAPSTDSEWRKQLSVDNAANMWVKMGCPKEKLVIGMPTYGRSFTLANTAKHGPNAPATGGGKEGIYTKEGGFLAYYEICEMLLNGAVYVWDDEMKVPYLVDGDQWVGFDDERAIRNKMQWIKTNGYGGAMVWTIDMDDFKGDVCGGNVKYPLIGAMREELLGISRGKEAKDVNWSEVAATFEDLEDEEEKPEPIKISVEEVLAKVRKPSKKHKIKSGLLASAQNTRPAQVFCYLTSWSAKRPGAGKFEPTNIDPKLCTHVVYAFATLKDHRLAENSDEDPDNYEQVIALRDVNPDLQILLAIGGWAFGSTPFKELTSNVFRMNQFVYEAIDFLRDYKFNGLDVDWEYPRGSDDRAAYVSLLRELRVAFEGEAKSSGLPRLLLTAAVPASFEAIAAGYDVTEISKYLDFINVMTYDFHGQWERTVGHNSPLFPLESTTGYQKKLTVDYSAREWVKQGAPKEKLLIGMPTYGRTFELINETQFDIGAPASGGGKPGKFTNEAGFLSYYEVCTFLAADNTTLVWDSEQQVPFAYRDNQWVGFDDERSLKTKMEWLKEQGFGGIMVWSIDMDDFSGRCGNGKYPLLNSLNDELKGYKVTLEYDGPYESRGPRGAYTTKDPHEVTCEEEDGHISYHKDWSDCTHYYMCEGERKHHMPCPANLVFNPQENVCDWPENVEGCHVPTEAPA

>SpCht8

MLDGGPDTILLRCDFLVIHHQCNVAANGQQQKKIICYHGSWSAMRTNLGKFRFETDMDPSLCTHLIYAFCGLQQNGELRIANHHLDLNLGNYKKFNNLKQKNPSLKTLLSVGGWTEGSLNFSIVAADPKKRATFLRSAIEILHKYNFNGLDIDWEYPNERHELKNNDRENFAVWLKELKVGFKPYGFLLTAAVKAISYQARQAYDIKEMVKQLDFINIMSYDLNGPWSEVVGIHSPLYAGPTDQDERAKQRNFNAIAQFWIRQGTPRHKIVMGIPFYGRSFTLKNSHNHSVGAAHNGSGIAGPYTKQPGIVGYNELCEMFKMKNSPWHLKWESKQMVPYAYYDNQWIGYDNEKSVALKVDYVNRHKLGGVMIWTVEMDDFRGVCGAKRYPLLRAINKGLRDIRSKNVKNNAQ

>SpCht10

MRELRGGLTAVTLLLLASFYTTFAHIRIDAPEEHHVQKTKPSFVRSAIESVPEDDTSSEHLSRLSMAPTFGDVFLPLRSAVESVPSVSTRSLESLRESSPLRHFVRDAVEAFPDDEDDEYIEEEEYSAAIRDSLVAEAMPENLDLDGPVPEPELDLEVENFQQPDAWSVSDKYSAFIEPYKKQNDIETLLPQPYRSDSPYQQLVKATEVKEIDSVVDVSKLREELKGYGALSNKRSREDLEFGFDNDEEDDEDIRPYGALAQIKATKPPRKNFNPSPEVLCYITNWAFYRKADGQFVPEYLKNKRLCTKLIYSFASLDPDHLNIKEFDPWVDIDNQFYSRTIETGIPVLIAMGGWTDSYGDKYSRLASDDIRRRVFASNLVGFLQRHGFSGLHLDWNYPKCWQSDCSKGPASDKPNLTKLLREIRSEFDRVDKKLKLGVAISGYKEIISEAYDFPELSKIVDYMTVMSYDYHGAWERQTGHVSPLYGRKGDKYPQYNTDYTMQLLLKLGAKKERLIMGIPFYGQTFTLERDSSQLIGEGTAAIGPGDAGEFTKQPGMLAYYEVCQRIRKQKWLTGRDPERKSGPYAMYRNQWVGYEDPASVEAKARYAVNAGFGGVSAWTVDLDDFQNRCCSESFPLLRAINRALGREDTEPPTRVNCARPPVPVTPIPPVMTTVSSDGSLGGGGMHEHTTANPVWQQPSTTASTTPKTSTVWWSPPSTTTTSTTTRRPTTTSTTSRRPTTTTTTRTTTTTRRPTTSTQKPTTIPAPAVVRPVVQASNCQPGEFYPDPYNCNAYYQCIVEGEIRQQFCPGGLHWNNQDKNCDWPASAKCTENKKPTNPTKAPPRRTTTTTSTTTRRPTQAPTKRPTTTTSSYWSTNRPSKPIATTRKPRPNKPNNNRPSMSARCNEGEYYSHRNCGQYYICINGALVPNSCGGNLHWDAVKKICDWPENVKCVTTKKYLRIVQSKGNPEDPCNGEERVPYPGDCSKYLFCLWNRLQAADCAPGLHFNAATGQCDWPESAKCNSDSNGGDTNDINPPKPKPAPTTARPTTTTIVCYFTNWAWYRKGLGRYTPDDINTDLCTHIVYGFAVLDYSNLILRTHDSWADIDNNFYTRVSGLKSKGVKVSLALGGWNDSLGDKYSRLVRNPQARARFVKHALEFLEKYGFEGLDLDWEYPVCWQTECNKGMPDEKEGFTALVRELSEAFVPRGLLLSTAVSPSKKIIDAGYDVPQLAKYFDWIAVMTYDFHGQWDKKTGHVAPLYYHPEDDYDYFNANYSLNYWIEKGAPSRKIVMGMPLYGQSFTLENTRNNGLNAKAPGPGKAGEFTRAAGFLAYYEICDRVKHQGWEVIQDEQGRMGPFARKGNQWVSFDDKAMIRKKSQLVRAMDLGGGMVWALDLDDFRNRCGEGIHPLLTQIHDVLKDPPSGYEPTPGLASPSEPESMEETVNSQESELENGSNASVESNPGDTEYIVDPNEDVEETDFEVVTSEHDFTEDTGPTGDEFKVVCYFTNWAWYRQGGGKFLPEDIDADLCTHIVYGFAVLNRQTLTIQPHDSWADLDNRFYERVADYRKKGTKVTVAIGGWNDSAGDKYARLVRSASARARFIRHVMEFIEKYGFDGLDLDWEYPVCWQVDCKKGTPDEKEGFADLVRELSEAFKPKGLLLSAAVSPNQKVIDAGYDVPQLTKYFDWIAVMAYDYHGQWDKKTGHVAPMYDHPEGTEGFNANFSINYWLEKGADRQKLVMGMPMYGQSFSLAQASDHGLNAKTYGGGEAGEATRARGFLSYYEICSYIRNKGWNVVRDARGRMGPYAFLRDQWVSFDDAPMIRHKSEYVKAMRLGGAMIWALDLDDFKNDCNCESYPLLKTINRVLRNYPGPHPNCVLEDKEKLMIAGTSSQPMGPPKPKPTAITHVEMAQTQSKPTKPTLAPMKMECGGKDYAPHEKDCNKYYICQYGELIEQKCPTGLHWNQNYCDWPQASQCMVRDDQTTHRPVVQRPKPTTTKAPPKTTKKPVAPPHKKPVARPKPKPTPAAPPLTGNEEYKVVCYFTNWAWYRPGQGKYVPEDIDENLCTHIVYGFAVLNSNALTIKTHDSWADIDNRFYERVVAYKKKGIRVTVAIGGWNDSLGSKYARLVLDPQARQRFIESVLAFCEKYGFEGLDLDWEYPVCWQVDCTKGSPAEKAGFASLVRELSAAFKPKGLLLSAAVSPSKKVIDAGYDVPTLARYFDWIAVMTYDFHGHWDKQTGHVAPLYYVEGDENPYFNGNYSINYWIEKGAPPQKLIMGMPLYGQSFSLADTHARSLNDKTIGPGRAGTFTRAGGFLAYYEICEQISNGGWTVIRDPQGRIGPYAYSGNQWVSYDDVNDIRRKSQFVRKMRLGGGMVWALDLDDFHGKCGCGKHPLLRTLNQELRGIPGQRANDCT

>SpCht11

MANYELIDETNGVRYKHIGWRIALMVSLCMCSTALVYRIWQEVYGPYYTVLAKYPHLKENIPTEWVDRAILYGSTYQNQTQESHDFRLVKNSTFRAKYLHFDQEDNSSLESNNILAPSPNETLMVCYYTISNAVLKSEELNLTQVNGTLCTHINVAQFPHLKFLLWIGGGGSASEGFPDMVKTHQSRKVFIQSLKDVLRTYKLDGCDLDWEFPSAYNRERMHFSQLLYEIRQEYVRERRPYLLSVAVAAPEGIALFAYDVGEINKYADHVNLMSYDYHFYSKATPFTGINAPLYARSNEHSILATLNINYSVNWWLKNGLDRKKLIVGLPTYGHTFTLVNPFNTAIGAPASGYGHTGQQGFISSSEVCWFLTKNVLSSLVFDKDACTCYASSGTEWMSFDQFMTISCKAKYIKMHELGGAMMFSLNTDDYKGICDPGRKFPLIGVVYRILKRV

>SpIDGF1

MLKINLKIYIFLIGLFCCQFASAVQTNKRLVCYYDVAAAGNHANLTEQSLMKALSRCTHLVYGFGQLVPRIFNVGHINLKPEPVSLKLKFPHLKIFLSVGGDKAATDVYMQLLEADKQQQNKFLKEIKNFLKVYHFDGLDLAFPFPRNKPRKVHSGMGLMLKRMKKMFTGDEIVDPNADKHKQQYTDLVTEMAKEFAKDKLNMSMTVLPNVNSTWYFDIKRIHKHFQFINLFAFDFLTPVRNREEADYTAALHYKSGPRRLEYANVEYQVSHWIRNGCPSQKLNLGIATYGQAWRMTVKSGLSGVPVIQNTDGPAKSSDPIRGVLSLPEICTKLKSKSLLQHKVIDEQRKYATYAFRPAKGKTQNGIWISFDDPEFAGFKAEYVKTNNLGGVALFDLQHDDFAAVCRQGTYPILKSVGRVLGVAKQVKK

>SpIDGF2

MKRVFASILAFLALLQISCAQQQPSKRVVCYYDSESSTRSGFAQLPQTDLEQGLEFCTHLIYGYAGLTRESFEIFSLNVDRDMFHYRQITALKAKFPQLKIYLSVGGDKDNDQVDPNKYIHFMEGGEPLYRNFIQSSINLLKTNGFDGIDLAFQFPRNKPRKVHSQIGMAWKKFKKLFTGNFIVDPDAETHKQQFTEFVGVMDEAYRIANLSMTLTVLPNVNSTWYFDIPAIKDKFEYVNLFAFDFLTPERNPEEADYTAPIYLKDEQNRLPHYNIDFQVQHWITNGCPANKLNLGIATYARTWKMTTDSELSGMPVVPGTVGPAEAGLQSKQEAGDFRGPNAPIRKVVDLERKYGNYAYRAADDNNEHGIWISFDDPDFAGIKTEYAKQKGLGGIALYDISYDDFRGLCTGARYPILRMVKSLGKLLNPDLEIALQFCSHLVYGYMGIKPLTHQVFSLHEDLDVHKHQFSEITALKRKFPHLKVLLSIGGDKDIDPGHPDKYLELLEGERVKQTAFINTAYTMVRTYGFDGIDLAYQFPKNKPLDPNADMHKEQFTTLIRDLRNVLKPDGLLLTLTVLPNVNSTWYFNVPVVSNFVDFVNLAAFDFLTPDRNPEEADYTAPLYELYDQNRLPHYNADYQVNYWLQHQCPAHKIILGMATYGRAWKMSSDSGTTGEPVVPTTEGPAEADRQSQTPGLLIWPEICYKLANSTNSFLKGANAPLRRVSELYGTYAYRPADSNGEHGIWVSYEDLESASNKASYVRSKGLGGLSFFDLSYDDFRGTCTGDRYPLLRTVKYRLWTYLIPETNLNKK

>SpIDGF3

MKFFLCVFLSLAFGQLALAANPNMVCFYDASSVDRDGLAKFSTPDFEIALQFCTHLIYGYAGINSENFHLKSLHNQRDIQRRHFAAITELKQKFPHVKFLLSVGGDKDFENPEKYVQLLEAGPEKYRAFAQSASELLRIYNFDGLDLAFQLPRNKPRKVHSGAGSAWKSFKKLFTGDFVVDEKADEHKEQFTNFVSTLKSVFNNDNFMITLTVLPNVNSTWYFNAPALAGHLDFVNLAAFDFTTPTRNPEEADYTAPLYPVPLEGNRLPHYNADFQVDYWISQRFPHNKLNLGIASYGRAWKMSSDSNSNGMPIVSETQGAADPGPQTKQGGLLDWAEICLKLPNPSNVGRSGAEAPLKRVADPTRKYGTYAFRPADANGEHGMWVSYEDPDTASNKAAYVKTRGLGGVALFDLSLDDFNGQCTGDKFPMLRAIKYRLL

>SpIDGF4

MRQSPSATVLKANITYAIGLAKFALSDLENALDFCTHLVYGYVGLNADTFRLATLNHERDVKNKQFVKVTALKEKFPYVKFLLGVGGDRDVGESEKYIKLLESGPEKQQAFIESARSLVRSLDFDGLDLAFQLPRNKPRKVHTGPGMAWKSFKKVFSGDFVVDPQASEHKEQFTELVKNLRKSFNENDLLLSLTVLPNVNSSWYFNAPALNNQIDFVNLAAFDFTTPDRNPFEADYTAPLYAPTIEGNRLSHYNVDYQVEHWTSQRFPKTKINVGIATYGRAWKMTLDSKDEGYPVVESTKGPAELATQTKTSGLLSWPEICLKLQPTKGVAQLRRFADPNRKHGTYAFRAADQSGDNGVWISYDDPEVAATKASYVANNGLGGVAVFDLSLDDFRGQCSTEKFPILKAIKYRL
